# Tagraxofusp in adult blastic plasmacytoid dendritic cell neoplasm: clinical trials and real-world outcomes: a systematic review

**DOI:** 10.3389/fimmu.2026.1853982

**Published:** 2026-06-08

**Authors:** Bassam Muthanna, Aadhila Abbas Manthiri, Leen Haj Saleh, Abdulrahman F. Al-Mashdali, Shehab F. Mohamed

**Affiliations:** 1 Department of Internal Medicine, University of Missouri-Columbia, Columbia, MO, United States; 2 College of Pharmacy, Qatar University, Doha, Qatar; 3 College of Medicine, Qatar University, Doha, Qatar; 4 Department of Hematology, National Center for Cancer Care and Research (NCCCR), Doha, Qatar

**Keywords:** blastic plasmacytoid dendritic cell neoplasm, BPDCN, capillary leak syndrome, CD123, hematopoietic stem cell transplantation, real-world evidence, systematic review, tagraxofusp

## Abstract

**Background:**

Blastic plasmacytoid dendritic cell neoplasm (BPDCN) is a rare, aggressive hematologic malignancy. Tagraxofusp, a CD123-directed cytotoxin, was approved based on phase I/II data, and subsequent studies have expanded evidence across trial and real-world settings.

**Methods:**

We systematically searched PubMed, Scopus, and Google Scholar for English-language human studies through January 25, 2026. We included adults (≥18 years) with BPDCN treated with tagraxofusp who reported efficacy and/or safety outcomes; abstracts without extractable data, narrative reviews, expert guidance, pediatric-only studies, and preclinical studies were excluded.

**Results:**

Twenty-seven studies were included, representing 343 unique adult patients treated with tagraxofusp after adjustment for overlapping analyses from the pivotal NCT02113982 trial program. Frontline trials reported overall response rates (ORR) of 71%–90% with CR/CRc rates of 56%–72%. Real-world cohorts showed ORR of 65%–90% and highlighted superior survival among patients bridged to allogeneic hematopoietic stem cell transplantation (HSCT). Capillary leak syndrome (CLS) was the defining toxicity, with variable incidence across clinical trials and observational cohorts.

**Conclusions:**

Tagraxofusp demonstrates consistent remission-inducing activity in adults with BPDCN across prospective and real-world settings. Long-term survival appears strongly influenced by successful bridging to HSCT. Evidence remains predominantly non-randomized, underscoring the need for comparative and combination studies.

## 1 Background

Blastic plasmacytoid dendritic cell neoplasm (BPDCN) is a rare and highly aggressive hematologic malignancy derived from precursors of plasmacytoid dendritic cells. It characteristically presents with cutaneous lesions, bone marrow involvement, and frequent dissemination to peripheral blood, lymph nodes, and visceral organs, and has historically been associated with poor clinical outcomes (1–4). Population-based analyses confirm its rarity, and multicenter series conducted prior to the advent of targeted therapy reported median overall survival of approximately 12–14 months with conventional chemotherapy approaches (1–4).

Because of disease rarity, prospective randomized trials have not been feasible, and earlier treatment strategies were largely extrapolated from acute myeloid leukemia (AML) or acute lymphoblastic leukemia (ALL) regimens. Although intensive chemotherapy could induce remission, relapse rates were high and durable disease control was uncommon without consolidation using allogeneic hematopoietic stem cell transplantation (HSCT) (5). Retrospective transplant analyses support HSCT, particularly when performed in first remission, as a key determinant of long-term survival (5, 6).

A defining biologic feature of BPDCN is uniform and high-level expression of CD123 (interleukin-3 receptor alpha chain), providing a rational therapeutic target (2). Tagraxofusp (SL-401; tagraxofusp-erzs) is a first-in-class CD123-directed cytotoxin composed of recombinant human interleukin-3 fused to a truncated diphtheria toxin payload. Upon binding CD123, the molecule undergoes internalization and inhibits protein synthesis, resulting in selective malignant cell death (7, 8).

Regulatory approval of tagraxofusp in 2018 was based on results from the pivotal phase I/II clinical trial (NCT02113982), which demonstrated clinically meaningful activity in both treatment-naïve and previously treated BPDCN (7). Subsequent expanded analyses, subgroup evaluations, and *post hoc* reports from the same trial have provided additional insights into durability of response and molecular risk stratification (9–11). Real-world cohort studies have further characterized treatment outcomes in routine clinical practice, while safety-focused reports have refined management strategies for key toxicities, particularly capillary leak syndrome (CLS) (12–16).

Despite these advances, the evidence base remains fragmented across heterogeneous study designs, overlapping trial analyses, patient populations, and reporting standards. Importantly, outcomes appear strongly influenced by whether patients can be successfully bridged to HSCT (5, 13, 14, 17). In addition, the relative contribution of tagraxofusp to long-term disease control, independent of transplantation, remains incompletely defined. While several narrative reviews have summarized evolving management strategies, no full peer-reviewed systematic review has comprehensively synthesized the efficacy and safety of tagraxofusp specifically in adult BPDCN. During our literature search, we identified only a conference abstract reporting a systematic review and meta-analysis of treatment outcomes in adults with BPDCN (Sandhu et al., 2023) (18), without a corresponding full manuscript available at the time of review.

Accordingly, this systematic review synthesizes adult (≥18 years) clinical trial, real-world cohort, and case-based evidence through January 25, 2026 to:

- evaluate efficacy across treatment settings;
- characterize the safety profile with emphasis on CLS and hepatotoxicity; and
- contextualize outcomes according to transplant eligibility and utilization.

## 2 Methods

### 2.1 Search strategy

We performed a systematic literature search of PubMed, Scopus, and Google Scholar for English-language human studies from database inception through January 25, 2026. Search terms included “blastic plasmacytoid dendritic cell neoplasm,” “BPDCN,” “tagraxofusp,” “tagraxofusp-erzs,” “SL-401,” “DT388IL3,” and “CD123-directed therapy.” Reference lists of included articles were manually screened to identify additional eligible studies.

Detailed database-specific search strategies, including full search strings, filters, and date limits, are provided in the Supplementary Material.

All retrieved records were imported into Zotero for citation management and initial deduplication. Following removal of duplicates, records were exported to Rayyan for title/abstract and full-text screening, where an additional duplicate review was performed to ensure accuracy of study inclusion.

**Figure 1:**
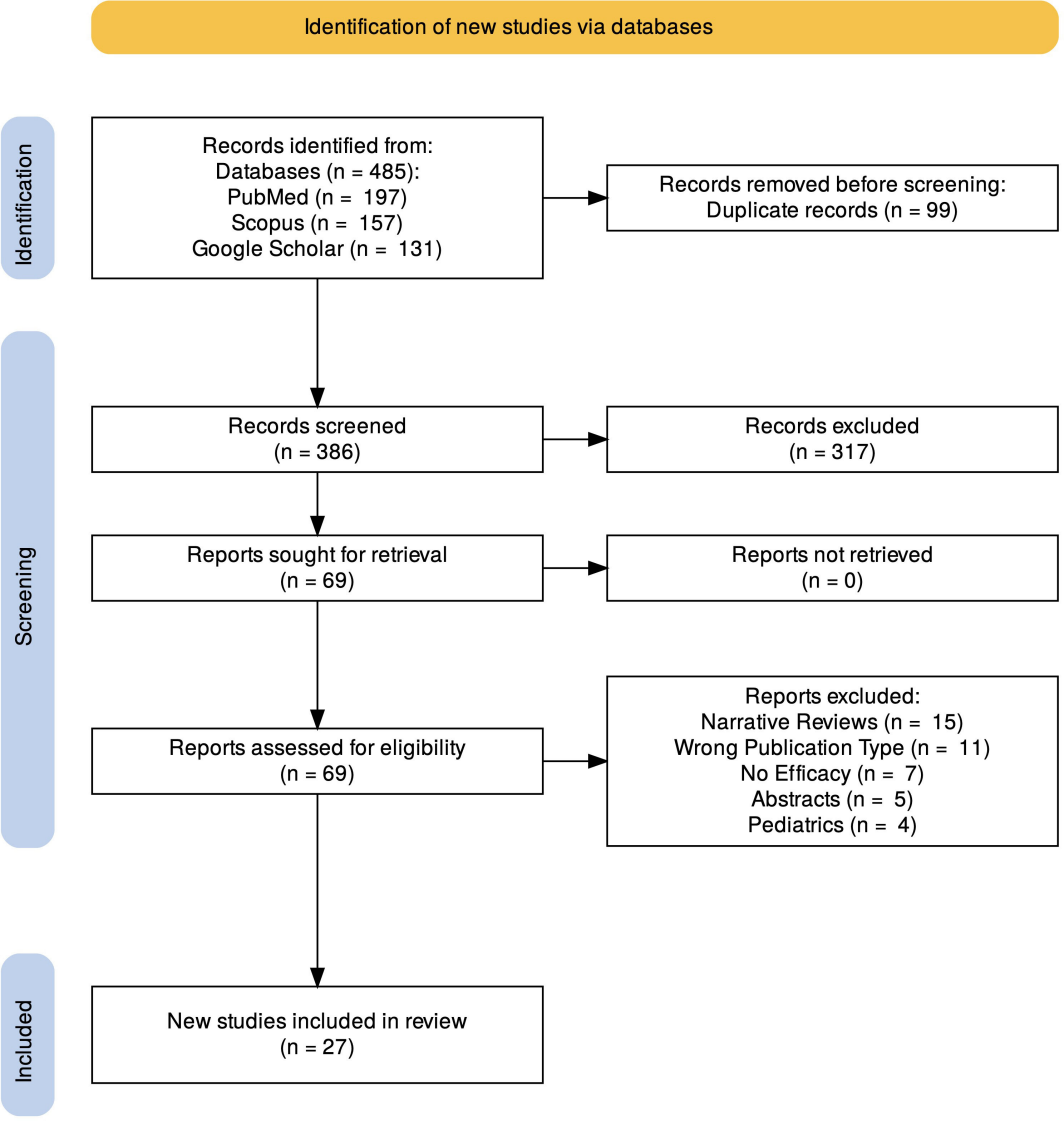
PRISMA flow diagram of study identification, screening, eligibility assessment, and inclusion for studies evaluating tagraxofusp in adult blastic plasmacytoid dendritic cell neoplasm (BPDCN).

This review followed PRISMA guidance; the PRISMA flow diagram is presented in **Figure 1** and the PRISMA checklist is provided in the Supplementary Material (19).

### 2.2 Eligibility criteria

We included studies that:

- Enrolled adults (≥18 years) with histologically confirmed BPDCN
- Evaluated tagraxofusp as monotherapy or in combination regimens
- Reported at least one efficacy or safety outcome
- We excluded:
- Abstracts without sufficient extractable outcome data
- Narrative reviews or expert guidance documents
- Pediatric-only studies
- Studies including mixed adult and pediatric populations without separate adult-specific outcome reporting
- Preclinical or mechanistic studies without clinical outcomes
- Duplicate publications

For the pivotal NCT02113982 trial, multiple publications reported overlapping patient populations at different time points or analytical focuses. The most comprehensive publication (Pemmaraju 2022; n=89) was used for primary efficacy and safety synthesis. Subgroup analyses (Pemmaraju 2025, poor-risk molecular features) and *post hoc* analyses (Konopleva 2026) were cited for specific findings but were not counted as independent trials to avoid double counting of patients.

### 2.3 Study selection and data extraction

Title and abstract screening were conducted using Rayyan, followed by full-text review of eligible articles.

Screening was performed independently by two reviewers, with disagreements resolved through discussion and involvement of a third reviewer when required.

Data were extracted using a standardized form capturing:

- Study characteristics (design, country, sample size)
- Patient demographics and disease characteristics
- Treatment details (line of therapy, monotherapy *vs* mixed regimen)
- Efficacy outcomes (overall response rate [ORR], complete response [CR], complete clinical response [CRc], survival metrics, and proportion bridged to HSCT)
- Safety outcomes (capillary leak syndrome [CLS], hepatotoxicity, hypoalbuminemia, treatment-related mortality, and discontinuation)

Response definitions were extracted as reported in the original studies. In BPDCN trials, CRc (complete clinical response) refers to complete remission with residual non-active skin abnormalities and does not imply incomplete hematologic recovery.

### 2.4 Quality assessment

Risk of bias and methodological quality were assessed using validated tools appropriate to study design:

- MINORS for non-randomized trials (20)
- Newcastle–Ottawa Scale (NOS) for cohort studies (21)
- Joanna Briggs Institute (JBI) checklist for case reports/series (22)

Detailed scoring and justification are reported in **Supplementary Table 1**.

### 2.5 Data synthesis

Given substantial clinical and methodological heterogeneity across study designs, treatment settings, and outcome reporting, quantitative meta-analysis was not performed.

Weighted pooling of response rates was not undertaken due to heterogeneity in study design, variation in sample size, and the presence of overlapping patient populations from the pivotal NCT02113982 trial program, which could introduce bias and overrepresent specific cohorts.

Instead, findings were synthesized descriptively across clinical trials, real-world cohorts, and case-based literature, with careful attention to overlapping patient populations in the pivotal NCT02113982 trial.

## 3 Results

### 3.1 Study selection

The literature search identified 485 records across PubMed, Scopus, and Google Scholar. After removal of duplicates and screening using Zotero and Rayyan, 69 full-text articles were assessed for eligibility. Twenty-seven studies met inclusion criteria (**Figure 1**).

Among these, six clinical trial publications were identified; however, four reports (Pemmaraju 2019, Pemmaraju 2022, Pemmaraju 2025 subgroup analysis, and Konopleva 2026 *post hoc* analysis) were derived from the same pivotal NCT02113982 study population. To avoid double counting, the most comprehensive dataset (Pemmaraju 2022; n=89) was used for primary efficacy and safety synthesis, while subsequent subgroup and *post hoc* analyses were cited for specific findings only.

Four real-world cohort studies and seventeen case reports/series were also included.

### 3.2 Study characteristics

A total of 27 studies met inclusion criteria, comprising 6 clinical trials, 4 real-world observational cohorts, and 17 case reports or small case series. Across these publications, 476 adult patients with BPDCN treated with tagraxofusp were reported. After adjustment for overlapping analyses from the pivotal NCT02113982 clinical trial program, 343 unique adult patients were identified for aggregate efficacy and safety synthesis.

Prospective trials predominantly enrolled treatment-naïve patients, although relapsed or refractory cohorts were also represented.

Real-world studies reflected broader routine clinical practice, including older individuals, patients with greater comorbidity burden, and a higher proportion of patients ineligible for transplantation.

Tagraxofusp was administered primarily as monotherapy in prospective trials, whereas real-world series occasionally reported its use within mixed therapeutic sequences consistent with contemporary management.

Median patient age across studies ranged from the mid-60s to early 70s, with a consistent male predominance.

### 3.3 Efficacy outcomes in clinical trials

Prospective efficacy outcomes are summarized in **Table 1**.

**Table 1:** Clinical trial efficacy outcomes of tagraxofusp in adult BPDCN.

Study (Ref)	Design	N	Setting	ORR (%)	CR/CRc (%)	Median OS (months)	HSCT (%)	Comments
Pemmaraju 2019 (7)	Phase I/II	47	29 TN; 18 R/R	90% (TN); 67% (R/R)	72% (TN)	NR (TN); 8.5 (R/R)	45% (TN)	12-mo OS 62%; 24-mo OS 52% (TN)
Pemmaraju 2022 (9)	Phase I/II update	89	65 TN; 24 R/R	75% (TN); 58% (R/R)	57% (TN)	15.8 (TN)	32% (TN)	24-mo OS 40%
Pemmaraju 2025 (10)	Subgroup analysis	65 TN	PCHM stratified	88% (PCHM); 74% (non-PCHM)	50% (PCHM); 58% (non-PCHM)	6.3 (PCHM); 18.9 (non-PCHM)	12.5% (PCHM); 35% (non-PCHM)	Inferior durability in PCHM
Frankel 2014 (8)	Phase I/II expansion	11	Mixed lines	78%	56%	NR	0%	First Clinial CD123 targeted therapy study.
Yokota 2026 (23)	Phase I/II Japan	11	7 TN; 4 R/R	71% (TN); 50% (R/R)	57% (TN)	NR	14% (TN)	No grade ≥3 CLS
Konopleva 2026 (11)	Post-hoc analysis	66	First-line subset	NA	57%	NR	NA	Post-hoc pivotal cohort

TN, treatment-naïve; R/R, relapsed/refractory; NR, not reached; NA, not available; CRc, complete clinical response; PCHM, poor-risk cytogenetic/molecular abnormalities.

The pivotal phase I/II clinical trial program (NCT02113982) represents the principal source of prospective efficacy data for tagraxofusp. The initial report by Pemmaraju et al. (2019) demonstrated high activity in treatment-naïve patients, reporting ORR of 90% and CR/CRc of 72%. This dataset was subsequently expanded in the updated analysis by Pemmaraju et al. (2022), which included 89 patients and serves as the primary efficacy reference for this review (7, 9–11).

In the updated analysis, frontline ORR was 75% with CR/CRc of 57%, and median overall survival reached 15.8 months among treatment-naïve patients, highlighting durable benefit primarily in patients eligible for subsequent transplantation.

In contrast, patients treated in the relapsed or refractory setting demonstrated ORR of 58%, with shorter survival durations than those observed in frontline therapy.

Subsequent analyses derived from the same clinical trial population further characterized outcomes. The Pemmaraju 2025 subgroup analysis, focusing on patients with poor-risk cytogenetic or molecular abnormalities, demonstrated comparable initial response rates but reduced durability of remission. The Konopleva 2026 *post hoc* analysis examined hematologic recovery patterns and transplant outcomes without introducing independent patient cohorts.

Additional prospective evidence includes the Japanese phase I/II study by Yokota et al. (2026), which reported ORR of 71% in treatment-naïve patients, confirming activity in an independent population. Earlier developmental experience reported by Frankel et al. (2014) demonstrated ORR of 78%, providing early clinical proof-of-concept evidence for CD123-directed therapy (8, 23).

Across prospective studies, a substantial proportion of responding patients proceeded to allogeneic hematopoietic stem cell transplantation (HSCT) following tagraxofusp induction. In the pivotal NCT02113982 trial program, approximately one-third to one-half of responders were successfully bridged to transplantation depending on treatment line and cohort definition. Similar transplant utilization patterns were observed in real-world cohorts, where HSCT was typically performed in patients achieving complete or near-complete remission after tagraxofusp therapy.

Across prospective trials overall, ORR ranged from approximately 71%–90% in frontline disease and 50%–67% in relapsed or refractory settings (**Table 1**).

### 3.4 Efficacy outcomes in real-world cohorts

Real-world efficacy outcomes are summarized in **Table 2**.

**Table 2:** Real-world efficacy outcomes of tagraxofusp in adult BPDCN.

Study (Ref)	Design	N	Setting	ORR (%)	CR/CRc (%)	Median OS (months)	HSCT (%)	Comments
Yun 2020 (17)	Retrospective cohort	49	Mixed regimens; TAG subgroup reported	NA	50% (TAG subgroup)	NR (overall); 12.1 (TAG subgroup)	NA	First-line outcomes by regimen; OS not reached overall; TAG subgroup OS reported
Lee 2025 (12)	Retrospective comparative	71	TAG n=39 vs VEN+HMA n=32	90% (TAG arm)	NA	NA	NA	12-mo OS 53% (TAG) vs 41.2% (VEN+HMA); ≥75y: 56.5% vs 38.1%
Herling 2025 (13)	Retrospective multicenter Cohort	26	First-line TAG	90% (evaluable)	65% (evaluable)	20.2 (overall); 37.0 (HSCT); 10.6 (no HSCT)	50% (13/26)	HSCT associated with longer OS; response-evaluable HSCT 55% (11/20)
Angelucci 2025 (14)	Retrospective Multicenter Cohort	24	Relapsed/refractory TAG	65% (evaluable)	40% (CR+CRi)	8.4 (overall)	42% (10/24)	Non-HSCT median OS 4.6; transplanted median follow-up 32.6; 24-mo continued response 40% overall

TAG, tagraxofusp; VEN+HMA, venetoclax plus hypomethylating agent; HSCT, allogeneic hematopoietic stem cell transplantation; NR, not reached; NA, not available; CRc, complete clinical response; CRi, complete response with incomplete recovery.

Four observational cohort studies evaluated the effectiveness of tagraxofusp in routine clinical practice across heterogeneous patient populations (12–14, 17). These studies included patients treated in both frontline and relapsed or refractory settings and frequently reflected broader clinical characteristics than those typically represented in prospective trials, including older individuals and patients with significant comorbidity.

Compared with clinical trial populations, real-world cohorts generally included older patients and those with greater comorbidity burden, as well as a higher proportion of individuals considered ineligible for transplantation. These differences likely contribute to variability in observed survival outcomes and underscore the importance of patient selection when interpreting real-world effectiveness data.

Across real-world cohorts, overall response rates ranged from approximately 65% to 90%, consistent with the activity observed in prospective clinical trials. Complete response or complete clinical response (CR/CRc) rates varied across studies but generally occurred in approximately one-half to two-thirds of responders, depending on patient population and treatment setting.

Median survival outcomes reported in real-world studies were variable. In cohorts where patients were successfully bridged to transplantation, median overall survival exceeded 30 months, whereas cohorts with limited transplant utilization reported shorter survival durations, frequently under 12 months.

A proportion of patients achieving remission after tagraxofusp therapy proceeded to allogeneic hematopoietic stem cell transplantation (HSCT). Similar to the pattern observed in prospective trials, HSCT was most commonly performed in patients achieving complete or near-complete remission. Reported transplant rates varied across cohorts but generally involved approximately one-third of responding patients, reflecting differences in transplant eligibility and institutional practice patterns.

Overall, the real-world studies demonstrate that response rates and transplant utilization patterns observed in routine practice are broadly consistent with those reported in prospective trials (**Table 2**).

### 3.5 Relapsed/refractory disease

In previously treated populations, ORR ranged from approximately 50% to 67% across trials and real-world series (**Tables 1**, **2**). Although response rates remained clinically meaningful, survival outcomes were inferior to those observed in frontline therapy. Nevertheless, tagraxofusp enabled successful bridging to HSCT in a subset of relapsed patients.

### 3.6 Safety outcomes in clinical trials

Safety outcomes reported in clinical trials are summarized in **Table 3**.

**Table 3:** Safety outcomes of tagraxofusp in adult BPDCN (Clinical Trials).

Study (Ref)	N	Population	CLS any-grade (%)	CLS grade ≥3 (%)	Hepatotoxicity (ALT/AST) (%)	Hypoalbuminemia (%)	TRM (%)	Comments
Pemmaraju 2019 (7)	47	Mixed (29 TN; 18 R/R)	18% (8/47)	4% (2/47)	ALT 64%; AST 60%	55% (26/47)	Reported (n=2)	Pivotal trial; CLS leading toxicity
Pemmaraju 2022 (9)	89	65 TN; 24 R/R	21% (19/89)	NA	ALT 64%; AST 60%	51% (45/89)	4.5% (4/89)	TRM: CLS (n=3) + MI (n=1)
Pemmaraju 2025 (10)	65	TN; PCHM stratified	0% (PCHM); 21% (non-PCHM)	NA (non-PCHM: ≥3 reported)	PCHM: AST 38% (all G3-4); ALT 25% (all G3-4); non-PCHM: ALT 56% (26% G3-4); AST 53% (25% G3-4)	PCHM 25%; non-PCHM 40%	Grade 5 events in non-PCHM (CLS/MI)	CLS absent in PCHM subgroup
Frankel 2014 (8)	11	Mixed lines	NA	NA	Transaminase elevation common; ≥3 in 5 pts	90.9% (10/11)	0%	Early study; limited grading details
Yokota 2026 (23)	11	7 TN; 4 R/R	54.5% (6/11)	0%	ALT 81.8%; AST 72.7%	54.5% (6/11)	0%	High CLS frequency but no severe CLS
Konopleva 2026 (11)	66	First-line subset (post hoc)	NA	NA	NA	NA	0%	Post hoc; safety not fully detailed

CLS, capillary leak syndrome; TRM, treatment-related mortality; TN, treatment-naïve; R/R, relapsed/refractory; PCHM, poor-risk cytogenetic/molecular abnormalities; G3–4, grade 3–4; NA, not available; MI, myocardial infarction.

The most characteristic toxicity associated with tagraxofusp is capillary leak syndrome (CLS). Across prospective studies, any-grade CLS occurred in approximately 18%–55% of patients, while grade ≥3 CLS occurred in 4%–33%. Variability in reported incidence likely reflects differences in monitoring protocols, diagnostic thresholds, and supportive management strategies across studies (7, 9, 10, 15, 16).

Hepatic transaminase elevations represented another commonly reported adverse event, occurring in approximately 60%–82% of treated patients. These abnormalities were generally transient and reversible with treatment interruption or supportive management. Hypoalbuminemia occurred in 51%–91% of patients across trial reports and has been described both as a pharmacodynamic effect of treatment and a potential risk factor for CLS development.

Additional adverse events reported across prospective studies included peripheral edema, weight gain, thrombocytopenia, and infusion-related reactions, although most were manageable with supportive care measures. Treatment discontinuation due to adverse events was reported in a minority of patients.

These findings suggest that toxicity is manageable within experienced centers using structured monitoring protocols.

Treatment-related mortality (TRM) was infrequently reported in clinical trials, reflecting improvements in patient monitoring, early recognition of CLS, and implementation of structured supportive care protocols during later phases of clinical development.

### 3.7 Safety outcomes in real-world cohorts

Real-world safety outcomes are summarized in **Table 4**.

**Table 4:** Safety outcomes of tagraxofusp in adult BPDCN (Real-World Cohorts).

Study (Ref)	N	Population	CLS any-grade (%)	CLS grade ≥3 (%)	Hepatotoxicity (%)	Hypoalbuminemia (%)	TRM (%)	Comments
Yun 2020 (17)	49	Mixed first-line regimens (TAG subgroup)	NA	NA	NA	NA	NA	Safety not primary endpoint; focused on survival by regimen and HSCT
Lee 2025 (12)	71 (39 TAG)	Comparative: TAG vs VEN+HMA	NA	NA	NA	NA	NA	Comparative effectiveness study; safety not detailed for TAG separately
Herling 2025 (13)	26	First-line TAG	50% (13/26)	23% (6/26)	38%	4% (isolated); 31% within CLS	4% (1/26)	Higher CLS vs trials; HSCT associated with improved survival
Angelucci 2025 (14)	24	Relapsed/refractory TAG	63% (15/24)	33% (8/24)	17–38%	33% (5/15 evaluable)	4% (1/24)	Higher severe CLS; infectious mortality reported

CLS, capillary leak syndrome; TRM, treatment-related mortality; TAG, tagraxofusp; VEN+HMA, venetoclax plus hypomethylating agent; NA, not available; HSCT, allogeneic hematopoietic stem cell transplantation.

Rates of CLS were comparable or slightly higher than in trials, likely reflecting inclusion of older and more comorbid patients in real-world cohorts.

However, severe CLS rates did not appear disproportionately elevated when structured monitoring protocols were implemented.

Hepatotoxicity and hypoalbuminemia patterns mirrored those seen in clinical trials.

### 3.8 Central nervous system involvement

Central nervous system (CNS) involvement represents a clinically challenging aspect of BPDCN management. Limited clinical data suggest that systemic tagraxofusp, when combined with CNS-directed strategies such as intrathecal chemotherapy, can achieve meaningful responses in selected patients (24). In the reported cases, durable remissions exceeding 24 months were observed, and one patient successfully proceeded to HSCT (7, 9, 13, 14, 17); however, systemic therapy was administered alongside intrathecal treatment, precluding conclusions regarding CNS activity of tagraxofusp as monotherapy. Given the absence of prospective data, optimal integration of CD123-directed therapy with CNS prophylaxis or treatment remains undefined and warrants further study.

### 3.9 Case reports and series

Seventeen case reports and small series collectively demonstrated that tagraxofusp can induce complete or partial responses in diverse clinical contexts, including treatment-naïve disease, relapsed/post-transplant relapse, CNS involvement, and transplant bridging scenarios. Although limited by small sample size and publication bias, these reports support the reproducibility of clinical activity and illustrate practical toxicity management in individualized settings (24, 26–40; **Supplementary Table 2**).

## 4 Discussion

The therapeutic introduction of CD123-directed therapy has meaningfully altered the treatment landscape of BPDCN, a malignancy historically associated with rapid relapse and limited survival despite intensive leukemia-based chemotherapy (1–4). The body of evidence synthesized in this review indicates that tagraxofusp consistently induces remission across frontline and relapsed settings, thereby increasing the proportion of patients who can proceed to potentially curative allogeneic hematopoietic stem cell transplantation (HSCT) (7, 9, 17).

A central observation across contemporary datasets is that long-term survival in BPDCN remains strongly influenced by successful transplantation. Population-level analyses and transplant-focused series demonstrate that durable remission is uncommon in the absence of allogeneic consolidation, particularly when HSCT is not performed in first remission (5). In the multicenter study by Yun et al., survival outcomes varied substantially according to first-line treatment strategy and transplant utilization, with improved long-term survival observed among patients undergoing HSCT compared with those managed without transplantation (17). Similarly, real-world tagraxofusp cohorts have demonstrated markedly superior survival among transplanted patients compared with non-transplanted counterparts (13, 14).

Collectively, these findings support the interpretation that tagraxofusp most frequently functions as a remission-induction and transplant-bridging strategy rather than as definitive curative therapy alone.

Clinical priorities differ according to transplant eligibility. In transplant-eligible adults, rapid achievement of deep remission with manageable toxicity is paramount. Prospective data from the pivotal NCT02113982 trial and its comprehensive update demonstrated high response rates in treatment-naïve patients, forming the evidentiary basis for frontline use (7, 9). In contrast, among transplant-ineligible or frail patients, therapeutic objectives may shift toward disease control and symptom mitigation. Real-world cohorts confirm that responses remain achievable in this setting, although survival is typically shorter when consolidation cannot be pursued (13, 14). These distinctions underscore the importance of early transplant evaluation and individualized therapeutic planning.

In relapsed or refractory disease, tagraxofusp retains clinically meaningful activity, although outcomes are inferior to those observed in previously untreated patients (9, 13, 14). The capacity to re-induce remission and permit delayed transplantation represents an important advance compared with historical salvage approaches, which were associated with limited efficacy and high relapse rates (1–4).

Comparative evidence remains limited and non-randomized. The retrospective study by Lee et al. suggested numerically improved survival with tagraxofusp compared with venetoclax-based therapy in older adults, particularly in patients aged ≥75 years (12). While these findings are clinically provocative, the absence of randomization and potential baseline imbalances preclude definitive conclusions regarding superiority. Venetoclax-containing regimens therefore remain important therapeutic alternatives rather than established replacements, and controlled comparative studies are warranted.

Biologic heterogeneity further refines therapeutic expectations. Subgroup analysis of patients with poor-risk cytogenetic or molecular abnormalities within NCT02113982 demonstrated that although initial responses may be achieved, remission durability and transplant transition rates are reduced in molecularly adverse disease (10).

Toxicity considerations remain central to safe administration. Capillary leak syndrome (CLS) is the defining adverse event of tagraxofusp, yet its reported incidence varies substantially across trials and real-world cohorts (7, 9, 10, 13–16). This variability likely reflects differences in baseline comorbidity, evolving clinician familiarity, monitoring intensity, albumin management protocols, and reporting thresholds (15, 16). Contemporary management algorithms emphasizing pre-treatment albumin optimization and early intervention appear to mitigate severity, even when minor manifestations remain common (15, 16). Importantly, treatment-related mortality has generally remained low across both clinical trials and observational cohorts (7–9, 13–16), supporting the feasibility of safe administration within structured monitoring frameworks.

Central nervous system involvement introduces additional complexity. Published case reports indicate that systemic tagraxofusp, administered alongside intrathecal therapy, can achieve meaningful responses in selected patients with CNS disease and may permit subsequent consolidation (25). However, these observations derive from limited case-based evidence, and prospective evaluation of optimal CNS-directed strategies is lacking. Standardized management approaches have not yet been established.

Despite encouraging activity across multiple study designs, the certainty of evidence remains moderate. Nearly all available data derive from single-arm trials or retrospective cohorts without randomized comparators. Overlapping analyses from the pivotal NCT02113982 study necessitate careful interpretation to avoid double-counting and to contextualize subgroup findings appropriately. Sample sizes remain small, follow-up duration is heterogeneous, and reporting standards vary across studies.

These limitations highlight the need for cautious interpretation of pooled outcomes and reinforce the importance of prospective, controlled studies.

Taken together, the accumulated evidence supports tagraxofusp as a highly active remission-induction therapy for adults with BPDCN, particularly when integrated into a transplant-oriented treatment strategy. Early transplant evaluation appears critical, as long-term survival is most consistently observed among patients who successfully undergo HSCT (5, 13, 14). Patients with adverse molecular features warrant heightened vigilance and consideration of investigational or combination approaches (10). Structured toxicity-prevention pathways, particularly for CLS, remain essential for safe implementation.

Future progress will likely depend on collaborative efforts capable of overcoming disease rarity. Controlled comparisons with venetoclax-based regimens, development of rational combinations, refinement of transplant timing, prospective evaluation of CLS prophylaxis, and incorporation of molecular risk stratification into treatment algorithms represent logical next steps.

## Conclusion

Tagraxofusp has emerged as a highly active remission-induction therapy for adults with BPDCN across clinical trial and real-world settings. While durable survival remains closely linked to successful bridging to allogeneic hematopoietic stem cell transplantation, consistent response rates across independent cohorts support its role as a central component of contemporary treatment strategies. However, the current evidence base is derived predominantly from non-randomized and single-arm studies, and comparative effectiveness data remain limited. Future collaborative efforts focusing on controlled comparisons, rational combination strategies, and refined transplant integration are likely to further clarify its optimal positioning in BPDCN management.

## Data Availability

The original contributions presented in the study are included in the article/**Supplementary Material**. Further inquiries can be directed to the corresponding author.
