# Supplementary material for: Tagraxofusp in adult blastic plasmacytoid dendritic cell neoplasm: clinical trials and real-world outcomes: a systematic review
Source: Front Immunol. 2026 Jun 8;17:1853982. doi: 10.3389/fimmu.2026.1853982 (PMC13284051; doi:10.3389/fimmu.2026.1853982)
Supplement: Supplementary file 2 [file Table2.docx]

**Supplementary Table S2. Summary of case reports and small case series of tagraxofusp in adult BPDCN.**

Abbreviations: BPDCN, blastic plasmacytoid dendritic cell neoplasm; ORR, overall response rate; CR, complete response; CRc, complete clinical response (CR with residual non-active skin abnormalities); OS, overall survival; Allo-HCT, allogeneic hematopoietic cell transplantation; CLS, capillary leak syndrome; NA, not available; NR, not reached/not reported (as stated in the source).

| **Study** | **N** | **Line** | **Regimen** | **ORR (%)** | **CR/CRc (%)** | **Median OS (mo)** | **Allo-HCT** | **CLS any** | **CLS ≥3** | **Key notes** |
| --- | --- | --- | --- | --- | --- | --- | --- | --- | --- | --- |
| Samhouri, Y 2020 | 1 | First line | Tagraxofusp monotherapy intitially, switched to Azacitidine + Venetoclax combination | 100% | NR | 14 months (Treatment ongoing) | 0 | 1(100%) grade 2 | 0 | TAG dose intolerability occurs in few patients, there is a need for alternative therapies (VEN +HMA) |
| Massone, C 2021 | 1 | first line | monotherapy | 100% | CR100% | ongoing | 1(100%) | 1(100%) | 1(100%) | CR achieved rapidly after first cycle Grade III CLS resolved with steroids, diuretics, albumin. Haploidentical HSCT successful, no GVHD. |
| Mouhayar, E 2021 | 1 | first line | Monotherapy | NR | NR | NR | 1 (100%) | 1 (100%) | 1 (100%) | Tagraxosusp proven to be effective, succesful Allo-HCT lead to complete remission. CLS is a major but manageable side effect of TAG; succesful rechallenge with appropriate prophylaxis is possible |
| Wang, S 2021 | 1 | Relapsed after allo-HSCT | combination | 1(100%) | 100% | NR | 1 before TAG | 0 | 0 | Well tolerated; no CLS or GVHD exacerbation; complete metabolic remission on PET-CT after 2 cycles |
| Gulati, R 2022 | 1 | First line | Monotherapy | 100% | 1(100%) | NR | 0 | NR | NR | Achieved CR with TAG; relapsed after 1.5 years with CD123-negative BPDCN clone; no safety data |
| Koerber, R 2022 | 1 | first line | TAG monotherapy | NR | NR( very good response initially but not durable) | 5 | 0 | 1(100%) | 1(100%) | Tagraxofusp induced initial strong response but discontinued due to toxicity. |
| Sibai, J 2022 | 1 | First line | monotherapy | 100% | 100% | 20 months(ongoing) | 0 | 1(100%) | 1(100%) | Achieved CR after only one induction cycle Long remission (>20 months) without HSCT.  Novel toxicities: BTS and gangrene requiring amputation. CLS and bicytopenia also occurred. |
| Vangala, D 2022 | 1 | first line | • TAG monotherapy • then high-dose methotrexate 4 g/m2 and ifosfamide (2 g/m2) as well as bi-weekly intrathecal therapy comprising methotrexate, cytarabine, and dexamethasone for Leptomeningeal disease • then allogeneic HSCT after conditioning with fludarabine (150 mg/m2), busulfan (6.4 mg/kg), and post-transplantation cyclophosphamide (100 mg/kg)2 | 100% | 100% | >10 months patient alive | 1(100%) | 0 | 0 | Tagraxofusp induced systemic CR; subsequent leptomeningeal relapse successfully treated with CNS-directed HD-MTX/ifosfamide + intrathecal therapy, autologous HSCT, then allo-HSCT |
| Andanamala, H 2024 | 5 | Mixed | Combination = 4/5, No therapy = 1/5 | NR | NR | NR | 2 (40%) | 1 (20%) | NR | TAG is feasible in real-world, elderly patients. CLS is a key toxicity. Allo-HSCT remains a critical consolidative option. BPDCN has high relapse rates |
| Dhakal, P 2024 | 1 | First line | •2 cycles of Tagraxofusp Monotherapy initially, then combination (triplet regimen: azacitidine 75 mg/m2 on days 1–7, venetoclax 400 mg daily on days 1–21 (with a ramp-up from 100 mgto 400 mgondays1–3in the first cycle), and TAG 12 lg/kg on days 4–6, administered in 28-day cycles. • Additionally, two doses of IT chemo for CNS involvement, (alternating between methotrexate 12 mg and cytarabine 70 mg twice weekly) | 100% | 1(100%) | ongoing | 0 | NR | NR | Some patients may relapse while on Tagraxofusp |
| Faustmann, P 2024 | 5 | first line | monotherapy | 60% | 2/5(40%) | 4 to 50months | 2/5(40%) | 3/5 patients (60%) | 1/5 (20%) | ORR 60% in elderly, comorbid cohort.  2 CRs (one ongoing >46 months, one 11 months).  Allo-HCT improved durability. CLS and hepatic toxicity common but manageable. |
| Pemmaraju, N 2024 | 2 | first line | Patient 1= Combination Patient 2: Monotherapy | NR | CR2(100%) | Patient 1: 32.5 Patient 2: 24 | 1 (50%) | 1 (50%) | 1 (50%) | Both patients achieved CR. The side effects werent discussed in depth, except manageable CLS in one patients |
| Pemmaraju, N 2022 | 4 | first line | monotherapy | 100% | 3/4(75%) | 15.8 months | 4/4(100%) | 3(75%) | NR | CR achieved rapidly (within 1 cycle in 2 cases). CLS common but manageable  All patients successfully bridged to allo‑SCT.  Durable remissions (up to 41 months). |
| Basir, Q 2025 | 1 | first line | induction: TAG used in combination with Hydroxyurea, mini-CVD , venetoclax (2 cycles) post-Allo SCT: TAG monotherapy | 1(100%) | CR1(100%) | >17 months | 1(100%) | 0 | 0 | CR achieved and sustained for >17 months. No CLS or high-grade toxicity.  GVHD manageable.  TAG maintenance feasible post allo-HCT. |
| Meier-Lienhard, R 2025 | 26 | Mixed | Mixed | NR | 16/26(62%) | 14.4 (95% CI: 0.8–6.2 years) | 7/26 (27%) upfront allo-HCT | 1/26 (3.8%) | NR | CR/CRc achieved in 16/26 patients Transplant patients had best survival (median OS 86 mo) Tagraxofusp induced CR in several but relapses common. Severe CLS occurred in one patient. |
| Staessens, S 2025 | 1 | first line | combination ( TAG + triple intrathecal chemotherapy (methotrexate (MTX), cytarabine, and methylprednisolone) + albumin prophylaxis) | 1(100%) | 1(100%) | NR | yes | NR | NR | TAG + IT chemotherapy for CNS involvemnet + Allo SCT resulted in excellent response in the 60 year old treatment naïve patients. This report does not discuss any side effects (safety parameters) |
| Brummer, C 2026 | 1 | first line | monotherapy | 1(100%)(forinitialtagraxofusptreatment) | 1(100%)[CRonlyforSkin] | >13 months (alive at last follow-up) | 0 | NR | 0 | TAG dose was modified to a lesser dose than usual due to fear of CLS in such an elderly patient with CKD and other comorbidities.  Even minimal, dose-reduced exposure to tagraxofusp (3 infusions) induced a durable 8-month response (CR of skin, but minimal residual infiltration in bone marrow after TAG)  Treatment was stopped due to acute kidney injury. At relapse, patient responded well to CHOP chemotherapy. |
